# Racial Disparities in Severe Cutaneous Adverse Reactions: A Report of Two Cases on Preventable Stevens-Johnson Syndrome/Toxic Epidermal Necrolysis Prescription Errors Through Medication Stewardship

**DOI:** 10.7759/cureus.101005

**Published:** 2026-01-07

**Authors:** Kritin K Verma, Merry A Mathew, Tramondranique Hawkins, Brooke Waltersheid, Dora R Goldstein, Sahil Kapur, Kermanjot Sidhu, Ethan J Matthew, Peter S Herman, Michelle Tarbox

**Affiliations:** 1 School of Medicine, Texas Tech University Health Sciences Center, Lubbock, USA; 2 Department of Dermatology, Texas Tech University Health Sciences Center, Lubbock, USA; 3 School of Medicine, Texas Tech University Health Sciences Center El Paso, Paul L. Foster School of Medicine, El Paso, USA; 4 Division of Dermatology, University of Toledo College of Medicine and Life Sciences, Toledo, USA; 5 Department of Medicine, Michigan State University College of Human Medicine, Ease Lansing, USA; 6 Department of Dermatology, Texas Tech University Health Sciences Center El Paso, El Paso, USA

**Keywords:** drug reactions, skin of color, socioeconomic status, stevens-johnson syndrome, toxic epidermal necrolysis

## Abstract

Stevens-Johnson syndrome (SJS) and toxic epidermal necrolysis (TEN) are severe, life-threatening cutaneous adverse reactions often triggered by medications. Common medications implicated in SJS and TEN include antibiotics, anticonvulsants, allopurinol, and nonsteroidal anti-inflammatory drugs (NSAIDs). Polypharmacy, particularly inappropriate or unnecessary medication use, increases the risk of severe adverse drug reactions. We present a case report discussing two patients with SJS/TEN secondary to non-indicated medication administration. The first patient was a 28-year-old male with antibiotic-related SJS after receiving amoxicillin-clavulanate for fungal balanitis. The second patient was a 54-year-old male with allopurinol-induced TEN despite no history of gout or hyperuricemia. These cases underscore the importance of medication stewardship, careful medication reconciliation, and clear communication between patients and providers.

## Introduction

Stevens-Johnson Syndrome (SJS) and toxic epidermal necrolysis (TEN) are rare, severe cutaneous reactions characterized by blistering and necrosis of the skin and mucous membranes, accompanied by systemic symptoms [1-4]. These conditions are present on a spectrum and are classified by the total body surface area (TBSA). SJS involves less than 10% of TBSA, while SJS/TEN overlap is defined by 10-30% TBSA, and TEN involves more than 30%.

SJS and TEN are delayed-type hypersensitivity reactions that involve a complex interplay between genetic and environmental factors [3,4]. Risk factors include exposure to medications such as beta-lactam antibiotics, anticonvulsants, non-steroidal anti-inflammatory drugs (NSAIDs), Nevirapine, allopurinol, and Nucleoside Reverse Transcriptase Inhibitors (NRTIs) [3,4]. Infectious agents, including HIV and *Mycoplasma pneumoniae*, have also been cited as clinical risk factors [2,3]. SJS/TEN management requires prompt identification and discontinuation of the offending agent and supportive care, while more severe cases benefit from specialized burn intensive care units [1,3,4].

Polypharmacy is defined as taking five or more prescription medications during the previous 30 days, and is associated with increased risk of adverse drug events, hospital admissions, falls, and cognitive impairment [5]. This is particularly concerning as the vast majority of adults older than 65 in the United States (US) routinely take more than five prescription medications, with prescription drug use and polypharmacy rates continuing to climb [5,6]. Polypharmacy is greatly complicated by incorrect dosing, administration, lack of medication reconciliation or interaction checks, and overprescription of non-clinically indicated medications for patient satisfaction [7].

Poor overall health status and growing patient volumes continue to place an undue burden on US providers and their clinical efficiency. As a result, side effect education and communication with patients often fall to the wayside, thus creating a perfect storm for polypharmacy and related adverse events, such as SJS and TEN [2]. Even further, racial and socioeconomic disparities coupled with genetic predispositions place Black and Asian patients at a higher risk of SJS/TEN and subsequent mortality [8]. Pharmacogenomic risk factors, including population-associated human leukocyte antigen (HLA) alleles implicated in severe cutaneous adverse reactions, further contribute to these observed disparities.

Many cases of SJS/TEN are preventable and arise in the setting of medication errors, including non-indicated prescribing, inappropriate antibiotic use, incorrect dosing, failure to reconcile medications, and inadequate monitoring for adverse reactions. Antibiotic stewardship failures, in particular, represent a critical and modifiable risk factor, as antibiotics remain among the most frequently implicated drug classes in SJS/TEN. This case report discusses two at-risk patients with severe cases of SJS/TEN following the administration of non-indicated medications. These cases emphasize the critical role of thoughtful prescribing, antibiotic stewardship, and proactive medication review in reducing severe cutaneous adverse reactions. Given the wide range of causative agents, clinicians should be aware of high-risk medications and remain vigilant in monitoring for potential adverse reactions.

## Case presentation

The authors obtained written consent from the patient for their photographs and medical information to be published in print and online, and with the understanding that this information may be publicly available. Patient consent forms were not provided to the journal but are retained by the authors.

Case 1

**Figure 1:**
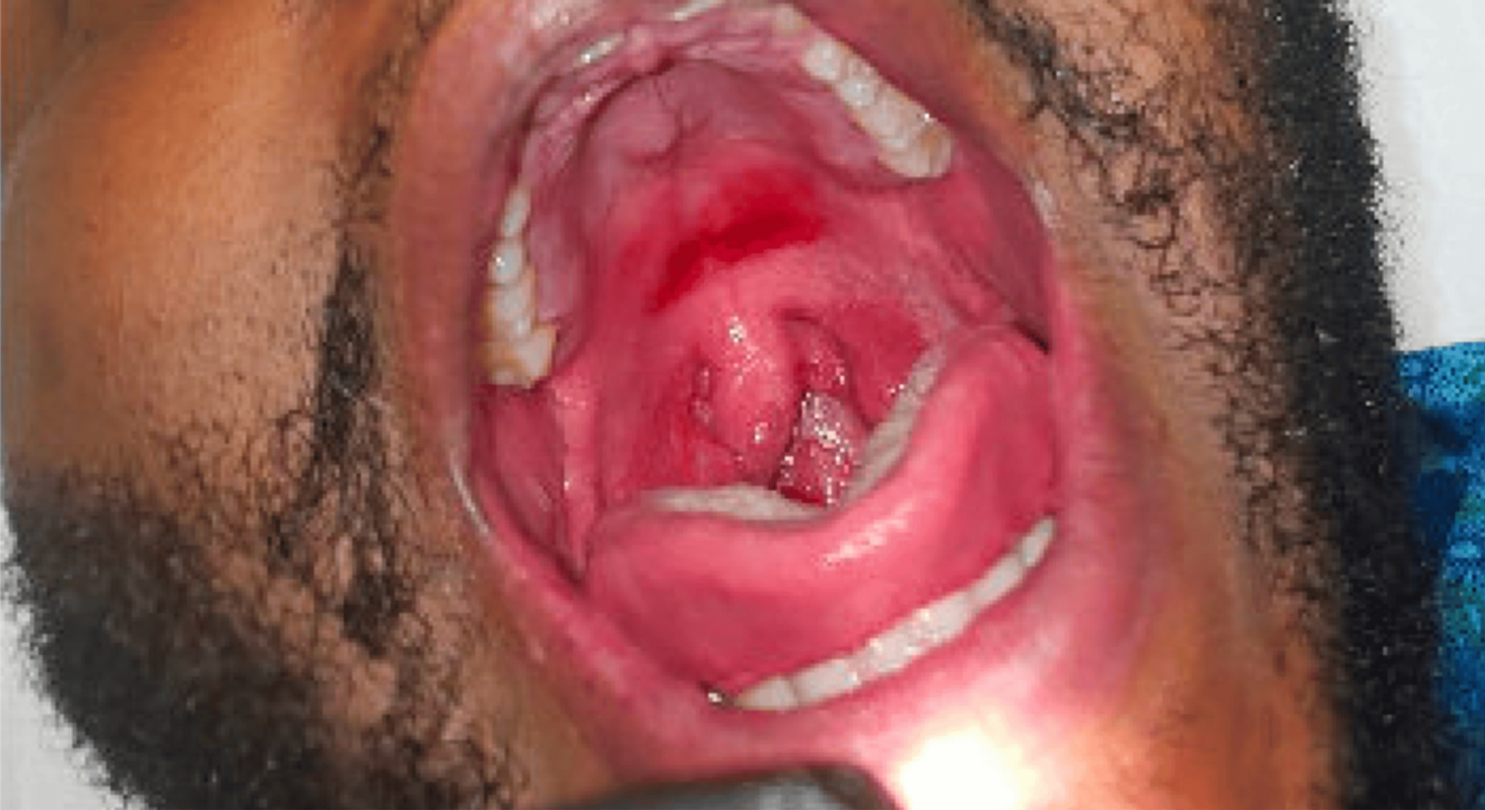
Case 1 - Oral mucositis in a patient with amoxicillin-clavulanate-induced SJS-TEN overlap syndrome. SJS-TEN: Stevens-Johnson syndrome - toxic epidermal necrolysis

A 28-year-old Black male presented with a one-day history of oropharyngeal swelling following three doses of amoxicillin-clavulanate, following diagnosis of acute fungal balanitis (Figure 1). His symptoms progressively worsened, with increasing penile pain and swelling, urinary hesitancy, and dysuria. Within hours, the palms and soles became exquisitely painful and swollen. Dusky macules with positive Nikolsky sign formed on the trunk while the lip mucosa started to slough, eventually progressing to involve the tongue and soft palate. A clinical diagnosis of SJS syndrome was made. The patient was managed with four days of intravenous immunoglobulin therapy, a single dose of systemic steroid (methylprednisolone 125 mg), and topical steroids (triamcinolone 0.1% BID, hydrocortisone 2.5% daily, and hydrocortisone 1% BID). Post-discharge, significant morbidity occurred, including one month of Foley catheter placement due to bladder spasms and eventual circumcision due to scarring.

Case 2

**Figure 2:**
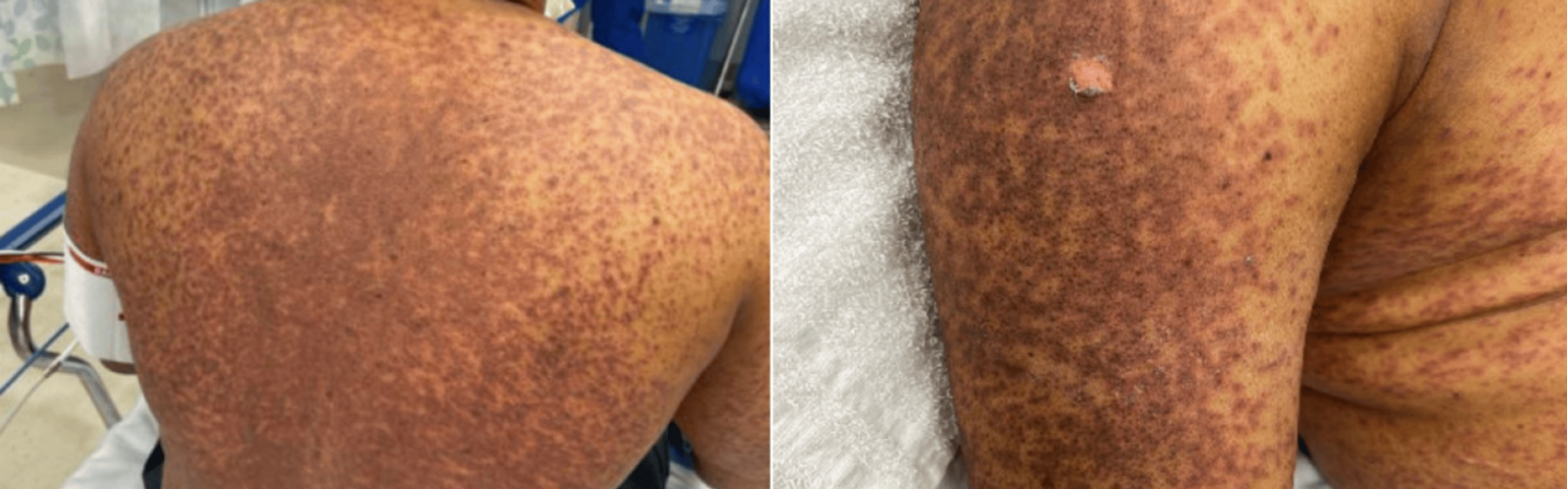
Case 2 - Diffuse rash of the posterior trunk and right upper extremity with positive Nikolsky sign in a patient with allopurinol-induced SJS SJS: Stevens-Johnson syndrome

A 54-year-old Black male presented with a two-day history of a rash involving the trunk, arms, legs, genitalia, palms, soles, and oral mucosa involving 50% TBSA; a positive Nikolsky sign was noted (Figure 2). Three months prior, he presented to his primary physician with a complaint of joint pain and was placed on allopurinol as a preventative measure, but had no history of gout or documented hyperuricemia. Clinical correlation with a punch biopsy revealing interface dermatitis with keratinocyte necrosis affirmed a diagnosis of TEN. Notably, this patient elected to independently stop his allopurinol upon observation of the initial truncal rash, substantially reducing his morbidity and mortality. The patient was managed with supportive care, including fluid resuscitation, pain control, and wound care (acetaminophen 1000mg q6h, acetaminophen-codeine 15 ml q6h, dexamethasone-diphenhydramine-nystatin-normal saline (Fred’s brew) 15 ml swish and spit QID). The truncal lesions healed with substantial post-inflammatory hyperpigmentation, which has persisted well past the one-year mark.

## Discussion

This case series highlights the importance of medication stewardship and awareness of potential side effects, even for commonly prescribed medications. As seen in our two patients, non-indicated administration of medications can lead to severe adverse reactions.

SJS and TEN are life-threatening reactions, and prescribers should familiarize themselves with the causative agents [2]. Previous studies have identified significant racial disparities in SJS/TEN, with Asian and Black patients having the highest incidence of SJS and TEN, with additional increased mortality, cost of care, and duration of stay compared to their white patient counterparts [2,8]. The hospitalization rate ratios for SJS/TEN were 11.9 for Asian patients and 5.0 for Black patients, compared to 1.0 for white patients [2,8]. Hispanic, Native American, and multiracial patients are also disproportionately affected [2]. Our case report describes two patients of color who developed SJS/TEN following exposure to medications that were either not clinically indicated or misprescribed, highlighting how lapses in medication stewardship may contribute to preventable, severe adverse drug reactions. For these patients, socioeconomic status, language barriers, and provider inexperience with skin of color may have led to poor patient outcomes.

The first case described a patient whose SJS led to long-term sequelae after receiving amoxicillin/clavulanic acid, not indicated for his diagnosis of balanitis. The second case involved a patient with no history of gout or hyperuricemia with allopurinol-induced TEN. A third case reported in the literature described a woman with a prior history of eosinophilia and systemic symptoms (DRESS) secondary to beta-lactams who was inadvertently re-prescribed beta-lactam antibiotics, precipitating another episode of DRESS [9]. This same patient later developed TEN secondary to sildenafil, which has been associated with SJS/TEN when administered in high doses [10]. Notably, this patient only spoke Gujarati, with translation provided by her son; therefore, this communication barrier may have contributed to beta-lactam administration despite her history of a previous drug reaction.

## Conclusions

Each case demonstrates the importance of thoroughly reviewing medication history and educating patients on indications and side effects. In patients with documented cutaneous drug reactions, avoiding re-administration is critical. As prescription errors continue to impact our US patient population, medication stewardship is paramount in preventing excessive adverse drug events. Prescription errors, including non-indicated prescribing and inadequate medication review, remain a significant and preventable contributor to adverse drug reactions.

Forward communication and a multidisciplinary approach to medication reconciliation may help decrease polypharmacy, while education and alterations in patient satisfaction score systems could prevent non-indicated prescriptions. Careful consideration of medication necessity, clear communication surrounding potential adverse effects, and prompt reassessment when new symptoms arise are necessary components of safe prescribing practices and can aid in reducing preventable medication-related harm. Together, these cases emphasize the importance of thoughtful prescribing and medication stewardship in minimizing preventable severe cutaneous adverse drug reactions.
